# Radiomics-Based Classification of Left Ventricular Non-compaction, Hypertrophic Cardiomyopathy, and Dilated Cardiomyopathy in Cardiovascular Magnetic Resonance

**DOI:** 10.3389/fcvm.2021.764312

**Published:** 2021-10-29

**Authors:** Cristian Izquierdo, Guillem Casas, Carlos Martin-Isla, Victor M. Campello, Andrea Guala, Polyxeni Gkontra, Jose F. Rodríguez-Palomares, Karim Lekadir

**Affiliations:** ^1^Artificial Intelligence in Medicine Lab (BCN-AIM), Departament de Matemàtiques i Informàtica, Universitat de Barcelona, Barcelona, Spain; ^2^Department of Cardiology, Hospital Universitari Vall d'Hebron, Barcelona, Spain; ^3^Vall d'Hebron Institut de Recerca (VHIR), Barcelona, Spain; ^4^CIBER-CV, Instituto de Salud Carlos III, Madrid, Spain; ^5^Departament de Medicina, Universitat Autonoma de Barcelona, Barcelona, Spain

**Keywords:** radiomics, machine learning, left-ventricle non-compaction, dilated cardiomyopathy, hypertrophic cardiomyopathy

## Abstract

Left Ventricular (LV) Non-compaction (LVNC), Hypertrophic Cardiomyopathy (HCM), and Dilated Cardiomyopathy (DCM) share morphological and functional traits that increase the diagnosis complexity. Additional clinical information, besides imaging data such as cardiovascular magnetic resonance (CMR), is usually required to reach a definitive diagnosis, including electrocardiography (ECG), family history, and genetics. Alternatively, indices of hypertrabeculation have been introduced, but they require tedious and time-consuming delineations of the trabeculae on the CMR images. In this paper, we propose a radiomics approach to automatically encode differences in the underlying shape, gray-scale and textural information in the myocardium and its trabeculae, which may enhance the capacity to differentiate between these overlapping conditions. A total of 118 subjects, including 35 patients with LVNC, 25 with HCM, 37 with DCM, as well as 21 healthy volunteers (NOR), underwent CMR imaging. A comprehensive radiomics characterization was applied to LV short-axis images to quantify shape, first-order, co-occurrence matrix, run-length matrix, and local binary patterns. Conventional CMR indices (LV volumes, mass, wall thickness, LV ejection fraction—LVEF—), as well as hypertrabeculation indices by Petersen and Jacquier, were also analyzed. State-of-the-art Machine Learning (ML) models (one-vs.-rest Support Vector Machine—SVM—, Logistic Regression—LR—, and Random Forest Classifier—RF—) were used for one-vs.-rest classification tasks. The use of radiomics models for the automated diagnosis of LVNC, HCM, and DCM resulted in excellent one-vs.-rest ROC-AUC values of 0.95 while generating these results without the need for the delineation of the trabeculae. First-order and texture features resulted to be among the most discriminative features in the obtained radiomics signatures, indicating their added value for quantifying relevant tissue patterns in cardiomyopathy differential diagnosis.

## 1. Introduction

Cardiomyopathies (CMs) are defined as primary myocardial disorders in the absence of other conditions that may affect the structural or functional properties of the heart's muscle (1). CMs are divided into distinct morphologic phenotypes (1), including hypertrophic cardiomyopathy (HCM) and dilated cardiomyopathy (DCM) as two of the most prevalent CMs. HCM is characterized by an increase in left ventricular (LV) wall thickness (2), DCM by LV (or biventricular) systolic dysfunction and dilatation (3), while both disorders are unexplained by loading conditions. Left ventricular non-compaction (LVNC) is a recently defined and poorly understood condition, characterized by prominent LV trabeculae, a thin compacted myocardial layer, and deep inter-trabeculae recesses (4).

Cardiac magnetic resonance (CMR) is current the gold standard imaging modality for the clinical assessment of CMs, as well as to identify and differentiate the different phenotypes. CMR is widely used in the diagnosis of HCM (5), DCM (6), and LVNC (7–9). However, some LVNC features can overlap with those of other CMs and LVNC patients might present with morphological findings of HCM and/or DCM (10). Furthermore, hypertrabeculation may also occur in the healthy population, which makes it challenging to differentiate physiologic from pathological hyper-trabeculation forms (11) by CMR. The difficulties to differentially and timely diagnose LVNC in clinical practice has motivated the development of new imaging indices, in particular, the Petersen (7) and Jacquier (8) coefficients, which estimate the level of hypertrabeculation in the LV myocardium. However, these coefficients, while they improve LVNC diagnosis (9), are challenging and tedious to estimate in practice, as they require expert and accurate identification and delineation of the trabeculae on the CMR images. This is a time-consuming task that is furthermore subject to inter-observer variability given the inherent complexity of the trabeculae.

Radiomics is an emerging image analysis technique for deeper phenotyping of cardiovascular health and disease in CMR (12). It enables the examination of a large pool of advanced imaging features that describe a wide range of complex, as well as subtle traits of the cardiac tissues at different scales and locations. Compared to existing cardiac indices such as those listed above, radiomics features encode multivariate information by capturing and combining heterogeneous morphological (e.g., sphericity, compactness) and appearance (e.g., entropy, coarseness) properties of the tissues. Hence, in the last years, several works have shown its potential for identifying new imaging signatures that can be leveraged for enhanced cardiac disease understanding (13, 14) and quantification (15–17). In addition to providing comprehensive indicators of cardiac health and disease, CMR radiomics features are easier to calculate as they only require the segmentation of the myocardial boundaries, and even this segmentation process can be automatized (18).

This work is the first to develop and evaluate a radiomics model for automatically differentiating LVNC, DCM, and HCM phenotypes in CMR. Based on a clinical dataset comprising different CM subgroups as well as healthy subjects from routine clinical practice, a machine learning pipeline is implemented to combine multiple radiomics features into a novel discriminative model of LVNC, HCM, and DCM. Subsequently, the obtained radiomics model is evaluated in great detail and its performance compared to the one obtained based on CMR indices, including the existing, manually estimated trabecular indices of LVNC. The results described in this paper show the promise of the proposed radiomics approach for achieving state-of-the-art LVNC, HCM, and DCM differential diagnosis more efficiently, while removing the need for the delineation of the LV trabeculae.

## 2. Data and Methodology

### 2.1. Dataset

The study cohort consists of 118 subjects, including 37 DCM, 25 HCM, and 35 LVNC patients, as well as 21 healthy control (NOR) subjects. HCM and DCM populations were available from the 2020 M&Ms MICCAI Challenge dataset (18). All patients for this study were assessed at the Hospital Universitari Vall d'Hebron (HUVH) following standard CMR protocols. Table 1 summarizes the clinical diagnostic criteria for each disease. In short, HCM, DCM, and LVNC diagnoses were established by expert cardiologists based on currently accepted imaging criteria (5–8) combined with other clinical data, such as electrocardiography, family history, and genetics. The mean age of the cohort was 49.4 ±17.97 and 76 subjects (65% of the cohort) were men (see Tables 2, 3 for more detailed information).

**Table 1:** Cohort size for each specific disease/control and clinical criteria for inclusion.

**Disease**	**Cohort size**	**Clinical inclusion criteria**
DCM	37	Depressed LVEF with increased LV volumes. Usually normal LV mass, wall thickness and asymmetry.
HCM	25	Increased LV mass with wall thickness >15*mm* and/or asymmetry >1.3. Ussually preserved LVEF.
LVNC	35	Jacquier ratio >20% and Petersen ratio >2.3. LVEF, LV volumes, LV mass and wall thickness can be normal or not.
NOR	21	Normal conventional CMR values. No history of relevant cardiovascular disease or systemic diseases.

**Table 2:** Cohort characteristics.

**Characteristics**	**Full cohort**	**DCM**	**HCM**	**LVNC**	**NOR**
Sample size	118	37	25	35	21
Age (years)	49.39 ± 17.97	50.24 ± 15.12	60.88 ± 17.84	44.57 ± 17.46	40.85 ± 16.99
Sex (M/F)	76 / 42	26 / 11	18 / 7	19 / 16	13 / 8
Male percentage (%)	64.41%	70.27%	72.00%	54.28%	61.90 %
BMI (*Kg*/*m*^2^)	26.37 ± 3.65	27.12 ± 3.89	26.32 ± 2.59	25.24 ± 3.45	25.67 ± 4.38
EDLVV (*ml*)	169.02 ± 63.88	225.40 ± 67.72	126.54 ± 34.58	159.07 ± 49.61	136.87 ± 24.47
ESLVV (*ml*)	97.03 ± 66.49	158.19 ± 75.45	51.34 ± 21.01	87.36 ± 45.56	57.65 ± 12.52
LV Mass (*g*)	122.29 ± 46.06	153.04 ± 51.70	139.05 ± 46.65	95.53 ± 26.37	102.52 ± 18.70
LVEF (%)	47.18 ± 16.03	32.47 ± 12.72	60.23 ± 9.79	47.26 ± 14.14	58.07 ± 4.09
Petersen coefficient	1.77 ± 0.90	1.61 ± 0.53	0.80 ± 0.39	2.86 ± 0.48	1.45 ± 0.54
Jacquier (%)	17.47 ± 10.08	16.70 ± 4.80	12.32 ± 2.72	24.30 ± 15.66	14.10 ± 3.19
ISV (*mm*)	11.15 ± 4.86	9.40 ± 1.70	19.12 ± 4.41	8.48 ± 1.61	9.09 ± 1.57
PW (*mm*)	7.26 ± 1.42	7.48 ± 1.46	8.25 ± 1.49	6.51 ± 1.10	6.95 ± 0.92
Assymetry (*IVS*/*PW*)	1.51 ± 0.57	1.27 ± 0.18	2.34 ± 0.75	1.30 ± 0.18	1.30 ± 0.16

*The table presents the average and standard deviation for the characteristics of the entire cohort, including age, sex, percentage of men, and BMI. On the right are presented the characteristics of each disease group*.

**Table 3:** Mann Whitney *U* statistical test for demographics and clinical characteristics group comparison.

**Demographics**	**DCM-HCM**	**DCM-LVNC**	**DCM-NOR**	**HCM-LVNC**	**HCM-NOR**	**LVNC-NOR**
Age (years)	0.011	0.08	0.05	0.0011	<0.001	0.26
BMI (*Kg*/*m*^2^)	0.354	0.144	0.208	0.378	0.415	0.432
**CMR indices**
EDLVV (*ml*)	<0.0001	<0.0001	<0.0001	<0.0036	0.08	0.031
ESLVV (*ml*)	<0.0001	<0.0001	<0.0001	<0.0001	0.028	<0.0014
LV mass (*g*)	0.02	<0.0001	<0.0001	0.0011	0.013	0.1214
LVEF (%)	<0.0001	<0.0001	<0.0001	<0.0001	<0.099	0.0005
Petersen coefficient	<0.0001	<0.0001	0.094	<0.0001	<0.0001	<0.0001
Jacquier (%)	<0.0001	0.014	0.019	<0.0001	0.040	0.0005
IVS (*mm*)	<0.0001	0.017	0.304	<0.0001	<0.0001	0.09
PW (*mm*)	0.014	0.0007	0.058	<0.0001	0.0006	0.046
Asymetry	<0.0001	0.11	0.12	<0.0001	<0.0001	0.4934

*P-values provided for existing CMR indices involved in classification. IVS, Inter-ventricular septum; PW, Posterior wall*.

### 2.2. CMR Clinical Indices

All patients underwent a standard CMR protocol. In brief, all scans were performed with a 1.5 Tesla scanner (Avanto, Siemens Healthcare, Erlangen, Germany), with typical cine parameters as follows: TR/TE (repetition time/echo time) = 3.2/1.5 ms, voxel size 1.4 ×1.4 ×8 mm, and a slice gap of 2.0 mm. The temporal resolution was interpolated to 25 phases per cardiac cycle (28–37 ms). The protocol includes a complete cine short-axis ventricular stack with the base to apex coverage acquired using balanced steady-state free procession (bSSFP) with one breath-hold per image slice. Short axis cine images were obtained and analyzed. Semi-automatic contouring of LV endocardial and epicardial end-diastolic (ED) and end-systolic (ES) borders was performed with Circle 42 (CVi 42) software (Calgary, Canada). A total of 9 existing CMR indices were quantified, including LV ejection fraction (LVEF), end-diastolic and end-systolic LV volumes (EDLVV, ESLVV), LV mass, inter-ventricular septum (IVS), posterior wall (PW) thickness, asymmetry (IVS/PW), and Petersen and Jacquier coefficients (7, 8). Right ventricle contours were not provided for the study; thus features were not considered. However, these diseases are predominantly related to the LV cavity, therefore missing information from the right ventricle was not considered relevant. Fractal dimensions (9) were not included in the experiments due to its limited applicability in daily clinical routine and its lack of prognostic correlation (19). Additionally, Petersen and Jacquier coefficients (7–9) were considered more validated for this experiments. All CMR analyses were performed by an expert cardiologist with several years of experience in the field.

### 2.3. Radiomics Extraction

From the region of interest provided by the LV endocardial and epicardial contours, radiomics were extracted from end-diastole (ED) and end-systole (ES) phases, following a pre-established pipeline from the open-source Python [(20), version 3.7.9] PyRadiomics library [(21), version 3.0]. A set of 420 radiomics features were extracted from the LV cavity (LV) and LV myocardium (LVMYO) within the original filter, including different types: 52 shape, 72 first-order, and 296 texture features (see Supplementary Material for a full list of radiomics extracted). We perform a radiomics features characterization by considering both the ED and ES phases to be able to identify disease-specific changes over a heartbeat cycle of the radiomics features.

Shape radiomics features quantify and measure the morphological traits in segmented contours, independently of the gray-level intensity distribution. These subset of features are easily interpretable, as they are closely related to surfaces and volumes calculated with existing CMR indices in clinical routine. Some of these features include simple metrics such as volume, elongation, or surface area, and more advanced metrics like sphericity.

First-order radiomics features are obtained from signal intensity characteristics based on the histogram. Specifically, first-order statistics describe the distribution of pixel/voxel intensities within the image region defined by the mask through commonly used and basic metrics, regardless of the spatial relationship (21). First-order radiomics features include easily interpretable features such as median or mean, and more mathematically advanced metrics such entropy, energy, or kurtosis.

Finally, texture radiomics features capture subtle changes in the gray-scale pixel distribution, identifying tendencies, and neighboring gray-scale changes through advanced matrix calculations. Texture features can be grouped in: Gray Level Co-occurrence Matrix (GLCM), Gray Level Size Zone Matrix (GLSZM), Gray Level Run Length Matrix (GLRLM), Neighboring Gray Tone Difference Matrix (NGTDM), and Gray Level Dependence Matrix (GLDM) (21).

Shape features are expected to capture morphological cardiac characteristics normally associated with each disease. On the other hand, both first-order and texture features are expected to play an important role in identifying grayscale changes in the LV or LVMYO tissue, a relevant trait of trabeculations in LVNC subjects.

### 2.4. Machine Learning Scheme

From the previous section, a total of 420 radiomics features were extracted and were potential candidates for inclusion in the targeted radiomics model for disease classification. However, not all of these features will have predictive power, and hence feature selection will be first applied to select the most optimal features for the classification task. We separated this feature selection process into 2 steps. First, we identify those that are highly correlated, for each feature, and remove them from the radiomics set as they carry a similar predictive signal. For this purpose, we estimated Pearson correlation between all features, and those above 0.9 were considered redundant. It is well-known that radiomics are highly redundant thus, with this first step we only aimed to remove the most correlated ones, and be further reduced with a more sophisticated feature selector. The procedure resulted in a reduction from 420 radiomics features to only 120. This reduced subset is introduced in the Pipeline function from Python Sci-kit learn package [(22), version 0.24.2] that has three different steps, including the additional feature selection method mentioned above:

1. **Normalization:** Data normalization is required before introducing the data into the machine learning models because different scales in the variables measured represent that features have different contributions to the model fitting, and this may introduce bias. We applied the MinMaxScaling function from the Sci-kit Python library (22). The StandardScaler was also considered, although no significant difference was found with a min-max scaling.
2. **Feature selection:** The number of features remaining was still large and had to be reduced before reaching the model building section. For this purpose, the SelectKBest function from Python's Sci-kit learn library (22) was performed. The algorithm works by selecting the best features based on univariate statistical tests. It selects the features according to two different parameters: highest *score function* and number of features (k). These parameters had to be defined beforehand. The score function parameter selected was *f_classif* , which computes the ANOVA *f*-value for the sample and provides the associated *p*-value for each feature based on the correlation with the class label. The *k* parameter is defined as the number of features selected by the feature selector. Prior the analysis, we do not know the optimal number of features to be selected, therefore the *k*-value before the analysis had to be defined within the range of parameters from 5 to 120 (i.e., number of possible features it may select), to be later introduced in the hyper-parameter optimization Grid Search CV. The number of features selected is tested iteratively, selecting the best k radiomics according to the score function and tested further with the model.
3. **Model building:** Three different machine learning algorithms were trained and tested: One vs. Rest Support Vector Machine (SVM), Multi-class Random Forest (RF), and Multi-class Logistic regression (LR) for classification (22). According to a recent review (23), both SVM and RF models were the most used techniques for conventional ML for image-based diagnosis. Additionally, prior knowledge from a recent publication (24) proved also the reliability of SVM and RF when dealing with radiomics. Finally, we decided to include Logistic Regression as one of the most-used techniques in statistical analysis for the purpose of increasing the comparison benchmark.

**Figure 1:**
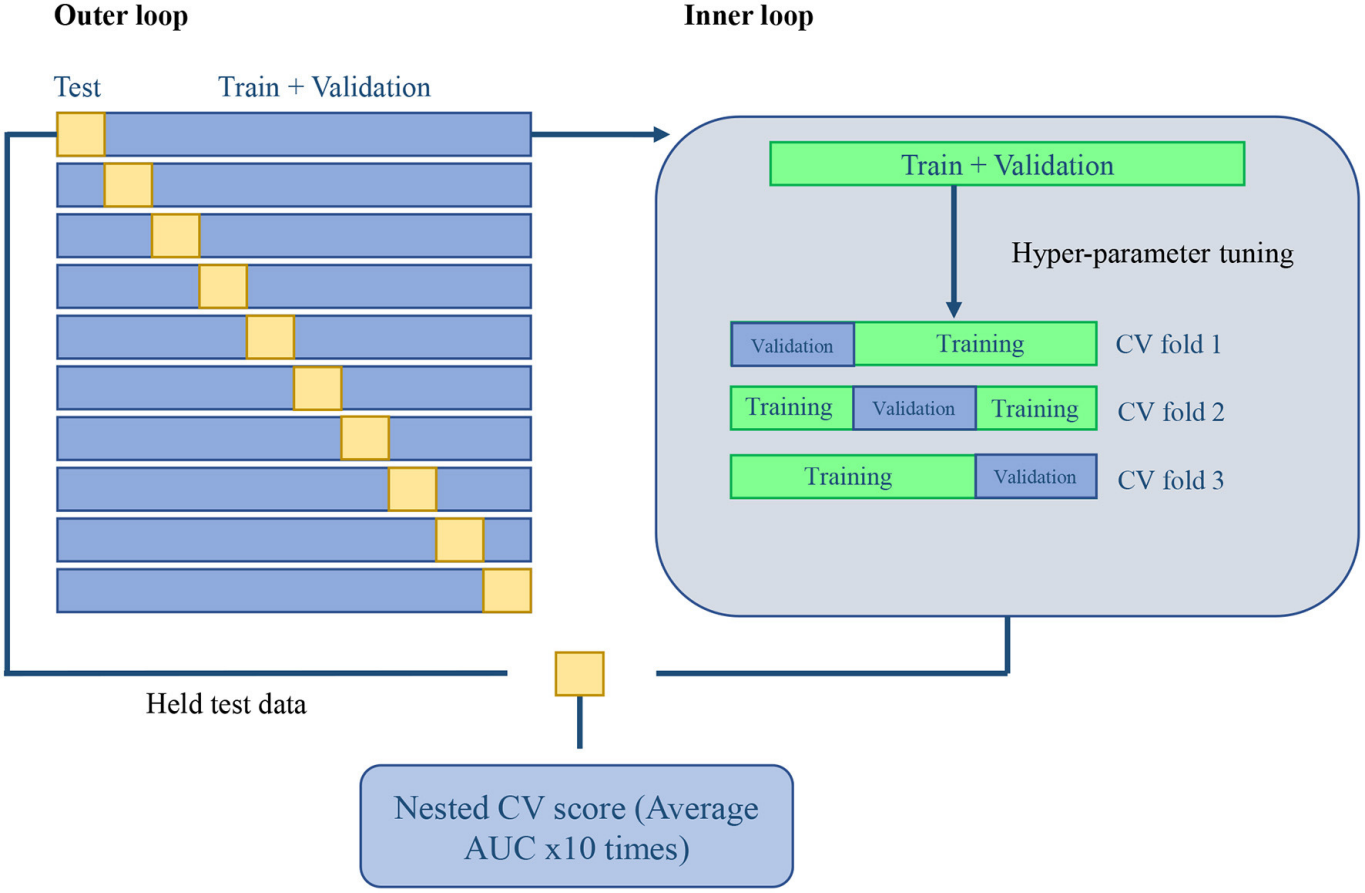
Machine learning validation: 10-fold outer loop Nested CV and a three-fold CV inner loop scheme.

For evaluation, the experiment is validated in a nested CV scheme (25). We performed a 10-fold outer loop and a three-fold inner loop (see Figure 1 for a more graphical description). This represents that for each fold in the outer loop, 90% of the data is kept for training and validation, while the remaining 10% will be held for testing. The same procedure was performed in the inner loop for each fold. The remaining train and validation data were split into 66% for training and 33% for validation. All the splits in our scheme were performed with Stratified K-fold sci-kit learn function (22) to keep classes balanced. The normalization and feature selection steps were performed in each fold of the nested CV scheme to avoid data leakage. This means that no knowledge of the held test set was introduced into the training stage, which could corrupt the learning process and its posterior generalization.

Models' performance are dependent on the hyper-parameters selected (26). For this purpose, a Grid Search CV (22) was applied in the inner loop, thus ensuring the optimal hyper-parameters were selected. Grid Search CV (22) is an optimizer algorithm that calculates the model's performance for each combination of hyper-parameters and keeps the one that achieved the highest prediction metric, to be later tested on the testing held data (see Supplementary Table 1 in Supplementary Material for the full list of hyper-parameters used).

Paired *t*-test on both distributions of testing AUC performances was performed to analyze the statistical significance for each general machine learning model across CMR indices and radiomics, as well as for each differential diagnosis (i.e., identifying a single disease class from the whole cohort) and prove they were comparable. Additionally, Receiver Operating Characteristic (ROC) curves were calculated to provide a better representation of the true and false-positive rates for each differential diagnosis. Due to the architecture of our Nested CV scheme, we obtain 10 different tests AUC, one per fold (10-fold outer loop, see Figure 1, left side). This means that each of the 10 models resulted from the Grid Search CV in the inner loop might be different (i.e., the combination of hyper-parameters and the number of features selected may vary depending on different characteristics of the training and validation set). Thus, to present the most relevant features on average in a single list, we analyzed the features selected by each of the 10 models and selected for representation those features that remained constant across the 10-folds and were finally sorted by feature importance score. With this, we create a highly approximated list of the most relevant radiomics features for each classification.

Since it is possible that different iterations selected a different number of features (i.e., for example, fold 1 could select 30 features and fold 2, 40 features), we analyzed the validation AUC values for each number of features (*k* = 10, 20, 30, 40) across all the combination of hyper-parameters to see the effect of increasing the number of features on AUC (see **Figure 3**). Finally, to provide a more clinical perspective, we analyzed the implications of feature type (shape, first-order, or texture), region (LVMYO, LV cavity), and phase (ED, ES) for each differential diagnosis, and linked them with the existing clinical knowledge.

## 3. Results

### 3.1. Classification Performance

In the first experiment, we evaluated and compared the performance of the different machine learning models (namely RF, OVR-SVM, and LR). As it can be seen in Table 4, the highest AUC values were obtained by the RF technique, for all models. Hence, the RF technique is used as the baseline models in the remainder of the experiments. Table 5 presents the in-depth results of the RF and each differential diagnosis.

**Table 4:** Summary table of the testing performance of the selected models.

	**CMR indices**		**Radiomics**		***p*-value**
**Models**	**Mean**	**STD**	**Mean**	**STD**
One vs. Rest SVM	0.972	0.03	0.942	0.03	>0.05
Random forest	**0.978**	**0.03**	**0.964**	**0.01**	>0.05
Logistic regression (multinomial)	0.970	0.03	0.956	0.03	>0.05

*The table provides AUC values for CMR existing indices and radiomics, along with the p-value. Bold values represent the highest AUC value obtained in test*.

**Table 5:** Generic and differential diagnosis AUC testing metrics for Random Forest model.

	**CMR indices**		**Radiomics**		***p*-value**
**General model**	**Mean**	**STD**	**Mean**	**STD**
Random Forest	0.978	0.03	0.964	0.01	>0.05
**Differential diagnosis models**					
DCM-vs.-Rest	**0.97**	**0.02**	0.93	0.03	>0.05
HCM-vs.-Rest	**1.00**	**0.00**	0.99	0.03	>0.05
NOR-vs.-Rest	0.95	0.02	**0.97**	**0.02**	>0.05
LVNC-vs.-Rest	**0.96**	**0.04**	0.92	0.03	>0.05

*P-values are presented in the table for statistical significance analysis and prove they are comparable. Bold values represent the highest AUC value obtained in test*.

**Figure 2:**
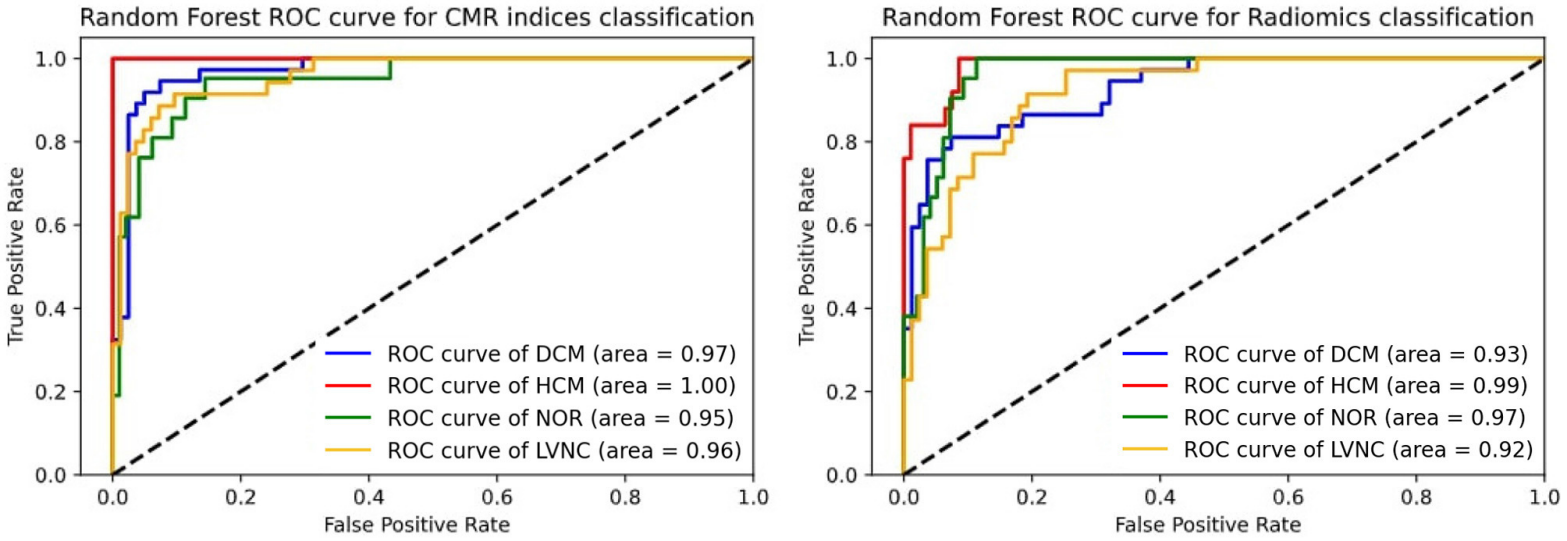
Roc curve comparison for differential diagnosis. The left subfigure represents the CMR indices ROC curve. The right subfigure represents the radiomics ROC curve.

Subsequently, we performed a comparison between the AUC scores obtained by the existing CMR indices (i.e., standard model) and radiomics models for classification. As it can be seen from Table 4, the radiomics model had a comparable performance to the standard model and there was no statistically significant differences between the two models (*p* >0.05). However, the radiomics model was obtained without the expert delineation of the trabeculae. In more detail, the ROC curves for the classification models are presented in Figure 2.

### 3.2. Radiomics Signatures

**Figure 3:**
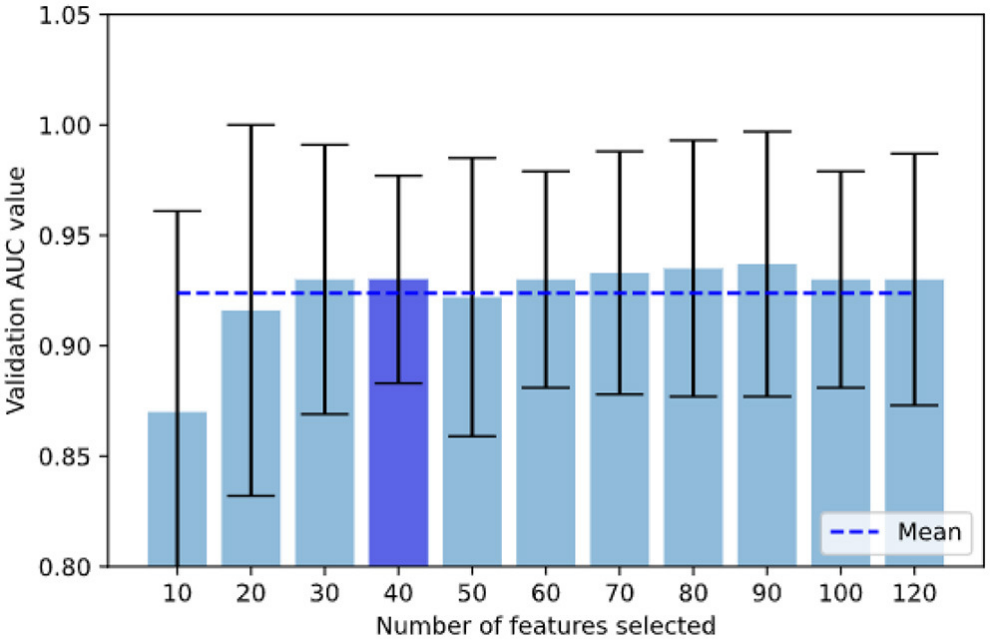
In our grid search scheme, we analyzed incrementally the number of features selected for all the hyper-parameter combinations and we show that validation AUC was not increasing significantly once reached 30–40 features, with the lowest STD at 40 features. AUC when selecting under 10 features reduced drastically.

In this section, we provide more details on the contributions of the different radiomics features to the classification models. We analyzed incrementally the number of features selected for all the hyper-parameter combinations and we showed in Figure 3 that the AUC values did not increase significantly after integrating 30–40 radiomics features in the model.

To further illustrate the predictive power of the radiomics features, Table 6 presents across subsections the 10 best performing radiomics for each differential diagnosis, sorted by their weighted feature importance (in percentage). Moreover, Table 7 shows the 10 best radiomics features involved for the general RF model.

**Table 6:** Top-10 best performing radiomics for each differential diagnosis, divided across subsection.

**Radiomics feature**	**Type**	**Region**	**Phase**	**Weight (%)**
**DCM vs. Rest**				
Minor axis	Shape	LVMYO	ES	7.0
Volume	Shape	LV	ES	6.7
Least axis	Shape	LVMYO	ES	5.8
Max2D diameter slice	Shape	LV	ES	5.5
Least axis	Shape	LV	ED	5.4
Least axis	Shape	LVMYO	ED	5.1
Long run high gray level emphasis	GLRLM	LV	ES	5.1
Minor axis	Shape	LVMYO	ED	5.0
Volume	Shape	LV	ED	5.0
Sphericity	Shape	LVMYO	ES	5.0
**HCM vs. Rest**				
Surface area to volume ratio	Shape	LVMYO	ES	7.4
Sphericity	Shape	LVMYO	ES	7.2
Large dependence high gray level E.	GLDM	LV	ES	6.9
Long run high gray level E.	GLRLM	LV	ES	6.7
Sphericity	Shape	LVMYO	ED	6.2
Skewness	First order	LV	ES	5.3
Gray level non-uniformity	GLSZM	LV	ES	5.1
Autocorrelation	GLCM	LV	ES	5.0
Energy	First order	LV	ES	4.9
Surface area to volume ratio	Shape	LV	ES	4.8
**NOR vs. Rest**				
Gray level non-uniformity	GLRLM	LV	ES	6.5
Run length non-uniformity	GLRLM	LVMYO	ES	6.0
Sphericity	Shape	LVMYO	ES	5.9
Low gray level run E.	GLRLM	LVMYO	ED	5.7
Dependence variance	GLDM	LVMYO	ES	5.7
Max2D diameter slice	Shape	LVMYO	ES	5.2
Gray level non-uniformity	GLSZM	LVMYO	ES	5.2
Long run high gray level emphasis	GLRLM	LVMYO	ED	5.2
Max2D diameter slice	Shape	LV	ES	5.0
Minor axis	Shape	LV	ED	4.9
**LVNC vs. Rest**				
Large dependence low gray level E.	GLDM	LVMYO	ES	6.6
Surface area to volume ratio	Shape	LVMYO	ED	6.5
Inverse variance	GLCM	LVMYO	ES	6.1
Long run emphasis	GLRLM	LVMYO	ES	5.9
Large area low gray level E.	GLSZM	LVMYO	ES	5.7
Large dependence low gray level E.	GLDM	LVMYO	ED	5.6
Large area emphasis	GLSZM	LVMYO	ED	5.2
Zone percentage	GLSZM	LVMYO	ED	5.1
Long run emphasis	GLRLM	LVMYO	ED	5.1
Percentile 90	First order	LVMYO	ES	4.8

**Table 7:** Top-10 best performing radiomics for RF model.

**Radiomics feature**	**Type**	**Region**	**Phase**	**Weight (%)**
**General RF model**				
Sphericity	Shape	LVMYO	ES	4.9
Long run high gray level emphasis	GLRLM	LV	ES	4.6
Sphericity	Shape	LVMYO	ED	3.6
Surface area to volume ratio	Shape	LVMYO	ES	3.6
Gray level non-uniformity	GLSZM	LV	ES	3.4
Minimum	First order	LVMYO	ED	3.4
Least axis	Shape	LV	ED	2.5
Large area low gray level emphasis	GLSZM	LVMYO	ES	3.0
Volume	Shape	LV	ED	2.9
Coarseness	NGTDM	LV	ES	2.2

*Please check Supplementary Table 2 in Supplementary Material for the full approximate list of radiomics selected*.

**Figure 4:**
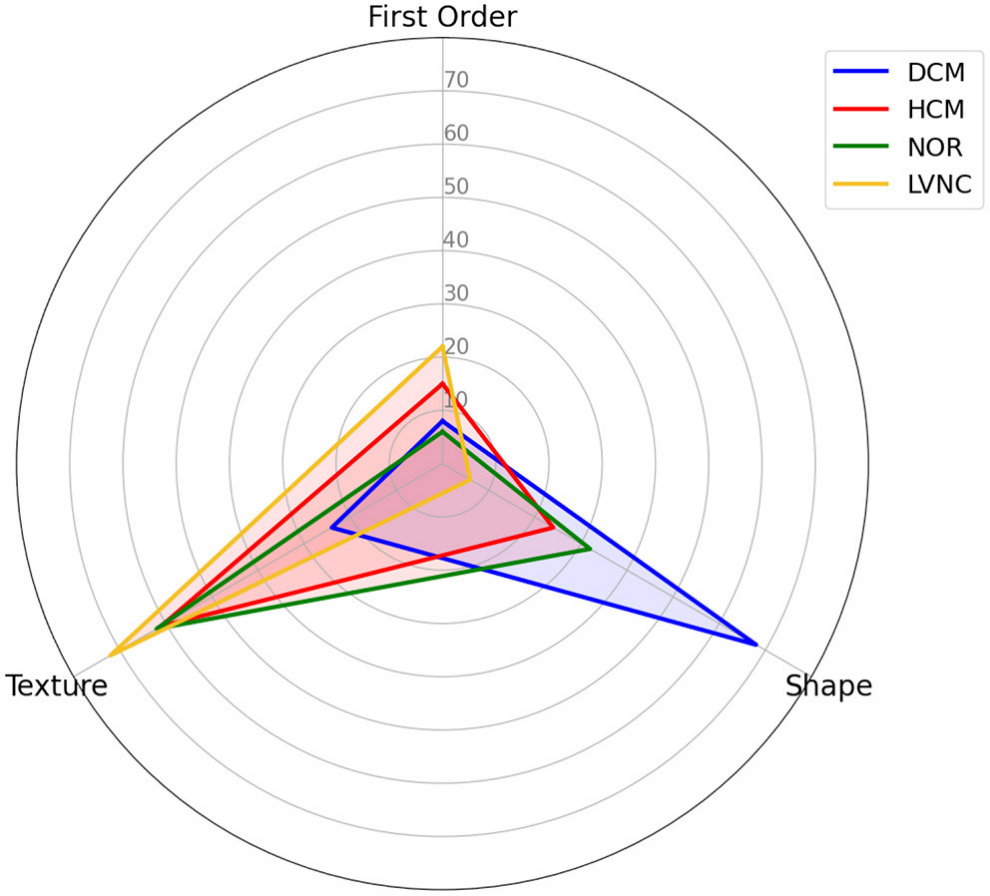
Differential diagnosis overlapping radar plot, comparing the distribution of the selected radiomics across types of radiomics.

By looking at Figure 4, we can observe that shape features play an important role when classifying DCM against the rest of the diseases. Alternatively, texture features have a higher impact in the classification of HCM and healthy subjects, and even a higher impact in the differential diagnoses of LVNC.

**Figure 5:**
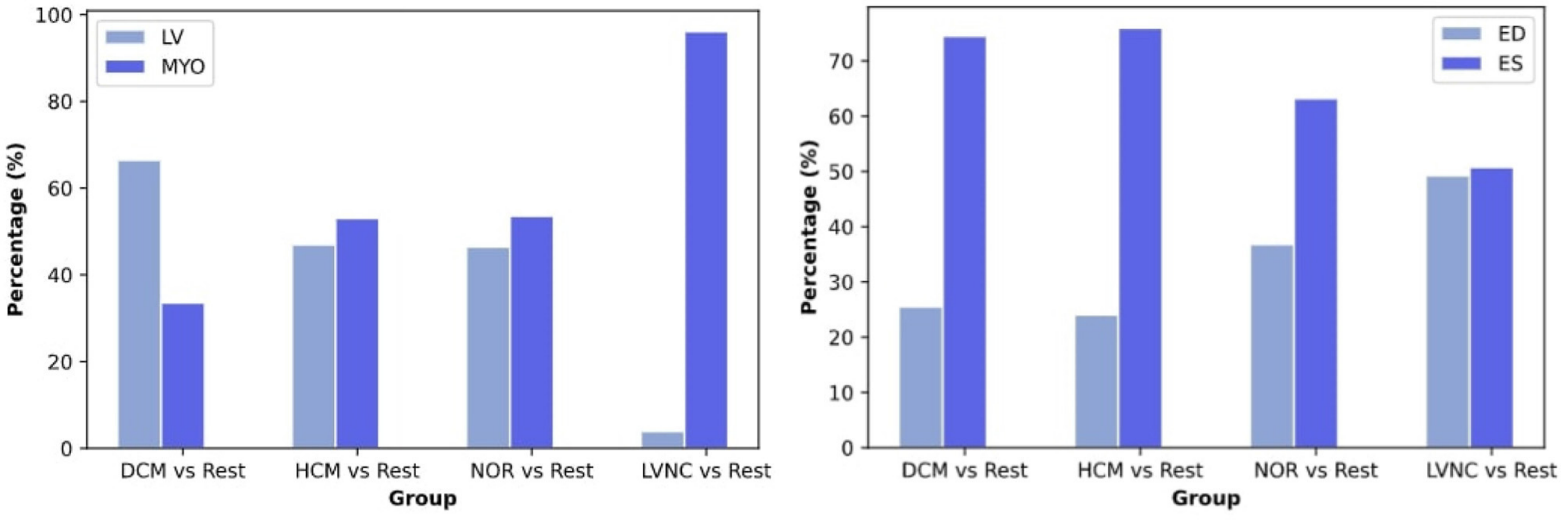
Distribution of the selected radiomics features across the cardiac structures [**left** image, i.e., LV myocardium (LVMYO) vs. LV blood pool (LV)] and cardiac cycle phase (**right** image). For this analysis, all the radiomics selected by the RF model were used.

Left side of Figure 5 shows the distribution of the selected radiomics features across the cardiac structures (i.e., LVMYO vs. LV cavity). For the identification of DCM, most of the radiomics features (65%) pertained to the LV cavity, while the remaining 35% belonged to the LVMYO. Conversely, features extracted from the LV cavity and the LVMYO participated equally to the identification of HCM and healthy subjects. Finally, the largest difference in terms of region importance can be found for LVNC, where almost 96% of the features belonged to LVMYO, with only a 4% to LV cavity (see Figure 5, left image). This last finding is in line with the existing clinical knowledge. Normally, papillary muscles are considered inside the LV cavity and not as a myocardial mass, according to the guidelines and clinical consensus among cardiologists. But in patients with LVNC conditions, trabeculae and papillary muscles are quantified [within Jacquier coefficient (8)] outside the LV cavity and included in the myocardial mass (LVMYO). For this reason, contrary to what the name itself suggests, the assessment between LVNC and the rest of cardiomyopathies is determined by changes or differences in the LVMYO.

Regarding the contribution of cardiac cycle phases, ES radiomics were more important than ED features for the classification of DCM, HCM, and healthy subjects. However, ES and ED phases play a similar role when assessing LVNC (see Figure 5, right image).

Finally, we compared the time needed to obtain the diagnoses using the standard as well as the proposed radiomics models. With the existing CMR indexes, our clinical experts spent approximately 9–12 min to delineate the trabeculae and then derive an LVNC diagnosis, on average. In contrast, for the proposed radiomics-based approach, the time to assess one subject was reduced to 10–20 s depending on the image characteristics [i.e., volume, slice images, or bin width (21)].

## 4. Discussion

### 4.1. Summary of Findings

Machine learning-based radiomics models showed excellent performance for differentiating between hypertrophic cardiomyopathy, dilated cardiomyopathy, left ventricular non-compaction, as well as healthy subjects. According to the results presented in Table 4 and Figure 2, radiomics and existing CMR indices resulted in similar performances.

The 10-most significant radiomics features for the general RF model comprise a combination of radiomics types, regions, and heart cycle phases (see Table 7). Looking in detail, myocardium sphericity seems to play an important role in the overall classification, occupying the first and third spots in terms of predictive power for the ES and ED phases, respectively. This can be explained by the remodeling of the heart that affects the global and regional structure of the left ventricle.

Moreover, texture features were found to add substantial information, positioning in the second top position of the ranking, which underlines the widely accepted diagnostic importance of myocardial tissue characteristics such as myocardial fibrosis (see Table 7). Specifically, Long Run High Gray Level Emphasis is a texture feature that explains longer contrasted gray level strings/regions, which can be related to the existence and prominence of myocardial trabeculations, typically associated with LVNC.

Regarding the radiomics features for each differential diagnosis, differences were found among the various diseases. Concretely, we can observe how the contribution of the radiomics features to the prediction are distributed, by type (Figure 4), region and phase (Figure 5). While shape features seem to play the most important role in DCM classification, texture features are more important when classifying HCM and LVNC subjects. This finding is in line with clinical knowledge: DCM is defined primarily by a dilation of the left ventricle, while myocardial fibrosis has a pivotal role in HCM diagnosis and LVNC is defined by the presence of myocardial trabeculations.

### 4.2. Limitations and Future Work

The findings presented in this paper must be considered in light of the study limitations, and future work may take different directions. Firstly, while the radiomics performance for automated diagnosis is promising, these results were obtained based on a single-center small-size clinical cohort. To confirm these promising results, future studies should be extended toward multi-center studies. Furthermore, this work relied on semi-automated, manually controlled, delineations of the LV endo and epi-cardial contours on the short-axis images, before the extraction of the existing indices and radiomics features. However, automatic segmentation of the ventricular boundaries has been extensively investigated using deep learning (18) and these models could be extended to segment pathological cases in particular for LVNC. Finally, this paper focused on the diagnosis of LVNC and related CMs. In clinical practice, subsequently, prediction of LVNC related events before they occur would enable early and personalized prevention. Our plan is to extend the proposed radiomics models to enable patient-specific risk prediction and prognosis estimation after LVNC diagnosis.

## 5. Conclusions

CMR radiomics constitutes a promising approach to differentially diagnose overlapping and complex conditions such as HCM, DCM, and LVNC. The classification performance of radiomics models are in-line with the one obtained by using existing CMR indexes but the diagnoses can be reached fully automatically without the need for expert delineation of the trabeculae as in previous works.

## Data Availability Statement

The original contributions presented in the study are included in the article/Supplementary Material, further inquiries can be directed to the corresponding author/s.

## Ethics Statement

Ethical review and approval was not required for the study on human participants in accordance with the local legislation and institutional requirements. Written informed consent for participation was not required for this study in accordance with the national legislation and the institutional requirements.

## Author Contributions

CI, GC, AG, JR-P, PG, and KL: design of work. CI, VC, CM-I, and PG: experimental setup and coding. CI, GC, and AG: interpretation of results. CI and GC: manuscript draft. VC, CM-I, GC, AG, JR-P, and KL: manuscript review. All authors contributed to the article and approved the submitted version.

## Funding

This publication was partially funded by the European Union's Horizon 2020 research and innovation euCanSHare project under grant agreement No 825903. KL received funding from the Spanish Ministry of Science, Innovation and Universities under grant agreement RTI2018-099898-B-I00. AG has received funding from the Spanish Ministry of Science, Innovation and Universities (IJC2018-037349-I).

## Conflict of Interest

The authors declare that the research was conducted in the absence of any commercial or financial relationships that could be construed as a potential conflict of interest.

## Publisher's Note

All claims expressed in this article are solely those of the authors and do not necessarily represent those of their affiliated organizations, or those of the publisher, the editors and the reviewers. Any product that may be evaluated in this article, or claim that may be made by its manufacturer, is not guaranteed or endorsed by the publisher.
