# Supplementary material for: Radiomics-Based Classification of Left Ventricular Non-compaction, Hypertrophic Cardiomyopathy, and Dilated Cardiomyopathy in Cardiovascular Magnetic Resonance
Source: Front Cardiovasc Med. 2021 Oct 29;8:764312. doi: 10.3389/fcvm.2021.764312 (PMC8586199; doi:10.3389/fcvm.2021.764312)
Supplement: Supplementary file 1 [file Data_Sheet_1.pdf]

Supplementary Material

1 SUPPLEMENTARY TABLES

Table S1. Hyper-parameters grid for optimization.

**Model**	**Hyper-parameter: [range]**
One-vs-Rest SVM	C: [0.01,1,10,100,1000]<br>gamma: [1,0.1,0.001,0.0001]<br>kernel = ['rbf']
Random Forest	bootstrap: ['True','False']<br>min_samples_leaf: [3, 4, 5, 6]<br>n_estimators: [100, 200, 300, 500, 1000]<br>min_samples_split: [8, 10, 12]<br>multi_class='ovr'
Logistic Regression	penalty: [l1]<br>Solver: ['liblinear']<br>multiclass = ['ovr']<br>max_iter = [100,150]
Select KBest	k: [5,10,15,20,25,30,40,50,60,70,80,90,100,110,120]

C: regularization parameter. RBF: Radial Basis Function kernel. OVR: One-vs-Rest.

Table S2. Full list of radiomics selected. This is an approximate listing, numbers may vary according to the figure 3

Radiomics	Type	Region	Phase	Weight
Sphericity	Shape	LVMYO	ES	4.9
Long Run High Gray Level Emphasis	GLRLM	LV	ES	4.6
Sphericity	Shape	LVMYO	ED	3.6
Surface Area to Volume Ratio	Shape	LVMYO	ES	3.6
Gray Level Non Uniformity	GLSZM	LV	ES	3.4
Minimum	First Order	LVMYO	ED	3.4
Least Axis	Shape	LV	ED	2.5
Large Area Low Gray Level Emphasis	GLSZM	LVMYO	ES	3.0
Volume	Shape	LV	ED	2.9
Coarseness	NGTDM	LV	ES	2.2
Skewness	First Order	LV	ES	2.8
InverseVariance	GLCM	LVMYO	ES	2.8
Skewness	First Order	LVMYO	ES	2.6
Surface Area to Volume Ratio	Shape	LV	ED	2.6
Percentile90	First Order	LVMYO	ED	2.6
Contrast	NGTDM	LV	ES	2.5
Large Area High Gray Level Emphasis	GLSZM	LVMYO	ED	2.4
Low Gray Level Run Emphasis	GLRLM	LVMYO	ED	2.4
Long Run High Gray Level Emphasis	GLRLM	LV	ED	2.4
Minimum	First Order	LV	ED	2.4
Cluster Shade	GLCM	LV	ES	2.3
Max 2Ddiameter Slice	Shape	LV	ED	2.2
Entropy	First Order	LVMYO	ED	2.2
Large Area Emphasis	GLSZM	LVMYO	ED	2.2
Large Area High Gray Level Emphasis	GLSZM	LV	ES	2.0
Minimum	First Order	LV	ES	2.0
Percentile10	First Order	LVMYO	ES	2.0
Large Area High Gray Level Emphasis	GLSZM	LVMYO	ES	2.0
Inverse Difference Moment Normalized	GLCM	LVMYO	ED	2.0
Surface Area to Volume Ratio	Shape	LVMYO	ED	2.0
Entropy	First Order	LVMYO	ES	2.0
Large Area Emphasis	GLSZM	LVMYO	ES	2.0
Elongation	Shape	LV	ES	2.0
Large Area Low Gray Level Emphasis	GLSZM	LVMYO	ED	2.0
Max 3Ddiameter	Shape	LV	ES	2.0
Zone Entropy	GLSZM	LVMYO	ES	1.8
Long Run High Gray Level Emphasis	GLRLM	LVMYO	ED	1.8
Low Gray Level Zone Emphasis	GLSZM	LVMYO	ES	1.8
Informal Measure of Correlation1	GLCM	LVMYO	ED	1.8
Least Axis	Shape	LVMYO	ED	1.8

Table S3. Full list of radiomics extracted. Note that for each radiomic feature, we obtain double (LV, LVMYO) and double (ED,ES). Therefore, only 105 are listed, a total of 420 (105x4) were extracted.

Type	Radiomics
Shape	Volume
	Surface Area
	Surface Area to Volume Ratio
	Sphericity
	Max3D diameter
	Max2D diameter Slice
	Max2D diameter Column
	Max2D diameter Row
	Major Axis
	Minor Axis
	Least Axis
	Elongation
	Flatness
First Order	Energy
	Total Energy
	Entropy
	Minimum
	Percentile 10
	Percentile 90
	Maximum
	Mean
	Median
	Interquartile Range
	Mean Absolute Deviation
	Robust Mean Absolute Deviation
	Root Mean Squared
	Skewness
	Kurtosis
	Variance
	Uniformity
GLCM	Autocorrelation
	Joint Average
	Cluster Prominence
	Cluster Shade
	Cluster Tendency
	Contrast
	Correlation
	Difference Average
	Difference Entropy
	Difference Variance
	Joint Energy
	Joint Entropy
	Informal Measure of Correlation1
	Informal Measure of Correlation2
	Inverse Difference
	Inverse Difference Normalized
	Inverse Variance
	Maximum Probability
	Sum Average
	Sum Entropy
	Sum of Squares
	Inverse Difference Moment
	Inverse Difference Moment Normalized
GLSZM	Small Area Emphasis
	Large Area Emphasis
	Gray Level Non Uniformity
	Gray Level Non Uniformity Normalized
	Size Zone Non Uniformity
	Size Zone Non Uniformity Normalized
	Zone Percentage
	Gray Level Variance
	Zone Variance
	Zone Entropy
	Low Gray Level Zone Emphasis
	High Gray Level Zone Emphasis
	Small Area Low Gray Level Emphasis
	Small Area High Gray Level Emphasis
	Large Area Low Gray Level Emphasis
	Large Area High Gray Level Emphasis
GLRLM	Short Run Emphasis
	Long Run Emphasis
	Gray Level Non Uniformity
	Gray Level Non Uniformity Normalized
	Run Length Non Uniformity
	Run Length Non Uniformity Normalized
	Run Percentage
	Gray Level Variance
	Run Variance
	Run Entropy
	Low Gray Level Run Emphasis
	High Gray Level Run Emphasis
	Short Run Low Gray Level Emphasis
	Short Run High Gray Level Emphasis
	Long Run Low Gray Level Emphasis
	Long Run High Gray Level Emphasis
NGTDM	Coarseness
	Contrast
	Busyness
	Complexity
	Strength
GLDM	Small Dependence Emphasis
	Large Dependence Emphasis
	Gray Level Non Uniformity
	Dependence Non Uniformity
	Dependence Non Uniformity Normalized
	Gray Level Variance
	Dependence Variance
	Dependence Entropy
	Low Gray Level Emphasis
	High Gray Level Emphasis
	Small Dependence Low Gray Level Emphasis
	Small Dependence High Gray Level Emphasis
	Large Dependence Low Gray Level Emphasis
	Large Dependence High Gray Level Emphasis
